# First evaluation of resistance to both a California OsHV-1 variant and a French OsHV-1 microvariant in Pacific oysters

**DOI:** 10.1186/s12863-019-0791-3

**Published:** 2019-12-12

**Authors:** Konstantin Divilov, Blaine Schoolfield, Benjamin Morga, Lionel Dégremont, Colleen A. Burge, Daniel Mancilla Cortez, Carolyn S. Friedman, Gary B. Fleener, Brett R. Dumbauld, Chris Langdon

**Affiliations:** 1 Department of Fisheries and Wildlife, Coastal Oregon Marine Experiment Station, Oregon State University, Hatfield Marine Science Center, Newport, Oregon USA; 2 Laboratoire de Génétique et Pathologie des Mollusques Marins, Ifremer, La Tremblade, France; 3 Institute of Marine and Environmental Technology, University of Maryland Baltimore County, Baltimore, Maryland USA; 4 Hog Island Oyster Co., Marshall, California, USA; 5 School of Aquatic and Fishery Sciences, University of Washington, Seattle, Washington, USA; 6 United States Department of Agriculture-Agricultural Research Service, Hatfield Marine Science Center, Newport, Oregon USA

**Keywords:** *Ostreid herpesvirus 1*, *Crassostrea gigas*, Heritability, Breeding

## Abstract

**Background:**

Variants of the *Ostreid herpesvirus 1* (OsHV-1) cause high losses of Pacific oysters globally, including in Tomales Bay, California, USA. A suite of new variants, the OsHV-1 microvariants (μvars), cause very high mortalities of Pacific oysters in major oyster-growing regions outside of the United States. There are currently no known Pacific oysters in the United States that are resistant to OsHV-1 as resistance has yet to be evaluated in these oysters. As part of an effort to begin genetic selection for resistance to OsHV-1, 71 families from the Molluscan Broodstock Program, a US West Coast Pacific oyster breeding program, were screened for survival after exposure to OsHV-1 in Tomales Bay. They were also tested in a quarantine laboratory in France where they were exposed to a French OsHV-1 microvariant using a plate assay, with survival recorded from three to seven days post-infection.

**Results:**

Significant heritability for survival were found for all time points in the plate assay and in the survival phenotype from a single mortality count in Tomales Bay. Genetic correlations between survival against the French OsHV-1 μvar in the plate assay and the Tomales Bay variant in the field trait were weak or non-significant.

**Conclusions:**

Future breeding efforts will seek to validate the potential of genetic improvement for survival to OsHV-1 through selection using the Molluscan Broodstock Program oysters. The lack of a strong correlation in survival between OsHV-1 variants under this study’s exposure conditions may require independent selection pressure for survival to each variant in order to make simultaneous genetic gains in resistance.

## Background

*Ostreid herpesvirus 1* (OsHV-1) is a widely distributed virus found to be the cause of Pacific oyster (*Crassostrea gigas*) mortality in oyster-growing countries of Europe, Australia, New Zealand, and the United States [1–5]. In Europe, Australia, and New Zealand, the OsHV-1 microvariants (μvars), which are distinguished from other variants by DNA sequence variations at two loci in the viral genome [6], are the most commonly found variants. OsHV-1 μvars were originally detected in France and are thought to cause greater mortality than other variants [5]; however, to our knowledge, an experiment quantifying the difference in survival of genetically similar oysters challenged by μvars and non-μvars under the same environmental conditions has yet to be performed in the laboratory. In the United States, OsHV-1 has been detected in adjacent northern California bays, primarily Tomales Bay [1, 7] and also Drakes Bay [8] and Bodega Bay (Burge, Moore, and Marshman unpub.).

Breeding for genetic resistance to OsHV-1 μvars in Pacific oysters has been successful in France and Australia. In France, four generations of mass selection starting from wild naturalized Pacific oysters resulted in an increase in OsHV-1 μvar survival in the field from 7 to 69% on average for three- to six-month-old selected oysters [9]. Mass selection consists of returning surviving oysters to the hatchery for spawning to create the next generation. This presents a considerable risk to hatchery operations if the hatchery is in a virus-free location, which is the case for hatcheries in the United States. In Australia, three generations of family-based selection starting from oysters previously selected for growth resulted in an increase in OsHV-1 survival in the field from 10 to 40% for one-year-old oysters for all families in the breeding program and from 40 to 85% for the top five families [10].

Laboratory screening methods for resistance to OsHV-1 μvars have been evaluated as an alternative to field screenings. Correlations between OsHV-1 μvar survival of oysters planted in three field locations in France and aquarium-held oysters injected intramuscularly with a purified virus ranged from 0.68 to 0.74 [11]. Similarly, genetic covariances ranging from 0.77 to 1.05 were found between field exposure to OsHV-1 and laboratory cohabitation with injected oysters in France [12]. Correlations between OsHV-1 survival of oysters planted over a period of three years in one field location in Australia and aquarium-held oysters exposed to OsHV-1 μvar ranged from 0.41 to 0.91 [10]. Further, correlations between OsHV-1 survival of oysters planted in two locations in New Zealand and aquarium-held oysters exposed to either a high or low concentration of OsHV-1 μvar ranged from 0.40 to 0.70 [13]. These results suggest that it is possible to use OsHV-1 μvar survival of oysters tested using laboratory assays as a proxy for OsHV-1 μvar survival in the field.

No oysters bred for OsHV-1 resistance to any variant are available in the United States. The rapid spread of the virus, as seen by the emergence of μvars in Europe in 2008 [5] and Australia and New Zealand in 2010 [2, 3], presents a significant risk to the US West Coast Pacific oyster industry. Restrictions on international and interstate oyster transportation currently exist in the US, but hypothesized modes of transmission other than oyster movement, such as ballast water and biofouling [14], limit the ability of these restrictions to prevent the spread of the Tomales Bay variant or μvars from other countries. Breeding oysters for resistance to OsHV-1 variants requires that the resistance be heritable, or under genetic control, in a breeding population.

Our objective in this study was to determine if the current oyster population at the Molluscan Broodstock Program (MBP), a United States West Coast Pacific oyster breeding program, has heritable variation in resistance to both the Tomales Bay variant (C-89) and a French μvar (C-25) of OsHV-1. As multiple OsHV-1 genotypes may be present in a particular location, the variant names will be accompanied by their “C” region genotypes as specified by Mineur et al. [15]. MBP has selected oyster families for improved yield since 1996 [16, 17]. There have only been two plantings of MBP families in Tomales Bay for the purpose of selection in October 1996 and 1999 [17]; therefore, a substantial level of resistance to mortality due to OsHV-1 in the breeding population was not expected.

## Methods

### Pacific oyster families

Seventy-five biparental oyster families were produced in February 2018 in the MBP hatchery at the Hatfield Marine Science Center (Newport, OR) according to previously reported methods [16, 17]. All oysters in a biparental oyster family are full siblings that were derived from one female and one male oyster. Seventy-one MBP families were selected on the basis of parental performance for yield, one family was a hybrid produced from two oysters inbred for three generations, two families had parents that naturally set in Willapa Bay, and one family was created for the purpose of commercial use by West Coast hatcheries.

Three French biparental oyster families and two French multiparental oyster families were produced in March 2018 at the Ifremer hatchery (La Tremblade, Marennes-Oléron Bay, France) according to previously reported methods [9]. Two biparental families were selected for resistance to OsHV-1 μvars, while one biparental family was selected for susceptibility to the virus. The two remaining families were unselected for their survival to OsHV-1 μvars and each was produced through a mass spawn of 30 oysters. The five oyster families were kept in a quarantine facility at the Ifremer station using UV-treated (40 mJ/cm^2^) seawater to prevent infection by OsHV-1 μvars, which are present in the adjacent Marennes-Oléron Bay.

### Plate assay under quarantine conditions in France

Two hundred and fifty, three-month-old randomly selected oyster spat from 69 MBP families and the hybrid family were placed in mesh bags and shipped on ice to the Ifremer Laboratory of Genetics and Pathology of Marine Molluscs (La Tremblade, France) on 21 May 2018. An authorization was obtained from the Directorate-General for Food of the French Ministry of Agriculture and Food prior to exporting the oysters from the United States. Spat arrived without mortality on 24 May 2018 and were placed in an aerated 120 L circulating aquarium at 20 °C with UV-treated and 50 μm sand-filtered seawater, addition of 17 μl of the antibiotic Flumisol® 36%/L, and no addition of microalgae. Flumisol® was used to prevent bacterial blooms. After 24 h in the aquarium, spat from each of the 69 MBP families, one hybrid family, and the five French families were placed in three six-well plates held in a room at 20 °C. Each well held five spat that ranged from 5 to 10 mm. For each family, two plates were used for the viral exposure treatment while the third served as a control. The viral exposure treatment involved adding 10 ml of OsHV-1 μvar-contaminated seawater to each well, while the control treatment involved adding UV-treated and filtered seawater to each well.

OsHV-1 μvar-contaminated seawater was prepared by placing thirty-five 11-month-old naïve oysters in a 20 L aerated aquarium with 17 μl Flumisol 36%/L and 50 g/L MgCl_2_ for two hours. Magnesium chloride (MgCl_2_) was used to induce gaping of oysters and facilitate injection of the virus suspension into their adductor muscles [18]. Each oyster was injected with 100 μl of 1000 DNA copies of OsHV-1 μvar/μl and returned to the aquarium with new seawater. The OsHV-1 μvar originated from tissue samples collected in 2016 from ten moribund oysters in the Marennes-Oléron Bay (France). The following day, oysters were removed from the aquarium, which held the contaminated seawater with a concentration of 2.2 × 10^7^ OsHV-1 μvar copies/ml. To determine the concentration, total DNA was extracted from the seawater using the QIAamp DNA Mini Kit (QIAgen) following the manufacturer’s protocol, and DNA quality and quantity were determined with a NanoDrop 2000 (Thermo Scientific). OsHV-1 μvar DNA was quantified by qPCR in duplicate using an Mx3000 Thermocycler (Agilent) following a protocol developed by Pepin et al [19]. Amplification reactions were performed in a total volume of 20 μl. Each well contained 5 μl of genomic DNA (5 ng/mL), 10 μl of Brillant III Ultra-Fast SYBR Green Master Mix (Agilent), 2 μl of each primer (5.5 μM: OsHV-1 DPFor 5′-ATTGATGATGTGGATAATCTGTG-3′, 5.5 μM OsHV-1 DPRev 5′-GGTAAATACCATTGGTCTTGTTCC-3′) [20], and 1 μl of distilled water. qPCR cycling conditions were: 3 min at 95 °C, followed by 40 cycles of amplification at 95 °C for 5 s, and 60 °C for 20 s.

Daily spat mortality counts were performed between three and seven days post-infection for the viral exposure and control treatments, with dead spat discarded after being counted. Spat were considered dead if they were gaping and did not close their shells upon physical agitation.

An additional three groups of spat were treated as the viral exposure treatment above for the purpose of viral DNA quantification over time. One group consisted of five families, which were randomly sampled among the MBP families, while the other two groups contained the French selected resistant and susceptible families. Spat from a well were sampled and pooled daily starting from the day of inoculation until the end of the experiment. DNA extraction and OsHV-1 μvar DNA quantification was performed as above with three replicates per group per day.

### Field trial in Tomales Bay, California

Four replicates of three-month-old randomly selected oyster spat, 5 to 10 mm in size, from 70 biparental families were planted in the outer bay of Tomales Bay (California, USA) on 23 May 2018 in a completely randomized design using the rack and bag method of oyster culture. The planted families included 64 out of the 69 MBP families tested in the plate assay, two additional MBP families, one hybrid family, two naturalized Willapa families, and one commercial family. A large 13 mm mesh grow-out bag (0.6 × 0.9 m) was used to hold four separate small 3 mm mesh replicate bags (0.3 × 0.3 m). Each small mesh bag held 35 oysters from a single family. The large bags were arranged in four rows and 20 columns at the same intertidal height. A HOBO™ temperature logger was placed in one of the large bags at planting. Oysters were checked biweekly for significant mortality. Due to resource limitations, all MBP families could not be planted in the field trial, and spat mortality counts, as well as DNA sampling for qPCR, were performed only once at the first observed significant mortality event, which was recorded on 1 August 2018.

OsHV-1-specific qPCR was used to determine the viral load in 31 live and 23 dead MBP oysters selected randomly in the field. The Zymo Quick-DNA Mini Plus kit was used following the manufacturer’s protocol to extract ~ 25 mg of gill and mantle tissue from each oyster. Using methods of Burge and Friedman [21] with some modifications, we targeted the OsHV-1 ORF 100/catalytic subunit of a DNA polymerase δ using the following forward (5′-TGATGGATTGTTGGACGAGA-3′) and reverse (5′-ATCACATCCCTGGACGCTAC-3′) primers and a standard curve from 3 to 3 × 10^7^ copies per reaction. Briefly, each 20 μl reaction included 10 μl of the Fast SYBR® Green Master Mix, 15 μg of BSA, 300 nM of each primer, and 2 μl of DNA. All standard curves were run in triplicate and samples in duplicate. All qPCR reactions were done using the Applied Biosystems 7500 Fast Real-time PCR system with a cut-off of 3 copies per reaction. Cycling conditions for each qPCR run were: 95 °C for 20 s followed by 40 cycles of 95 °C (3 s) and 60 °C (30 s). Following each run, a melting curve analysis was performed to confirm the specificity of each qPCR reaction by comparing the melting temperature peak of the positive control DNA to that of the experimental samples. Additionally, to confirm amplifiable DNA in all samples, we targeted the Pacific oyster 18S gene using the primers CG 18S F 5′-CAGCGAAAGCATTTGCCAAG-3′ and CG 18S R 5′ CACCCA CCG AAT CAA GAA AGA G 3′ [21]. Reaction conditions were the same as above; however, cycling conditions were: 95 °C for 20 s followed by 40 cycles of 95 °C for 3 s and 55 °C for 30 s. A melting curve analysis was performed after each run as above.

### Statistical methodology

Genetic selection for disease resistance is successful only when genetic variation exists for a resistance phenotype, which was cumulative percent survival for each plate containing a single family in the plate assay and percent survival for each small bag containing a single family in the field trial. A Bayesian Gaussian process regression model [22] was used to estimate the genetic and error variances in order to estimate heritability and its uncertainty. A high heritability means that closely related families are more similar in their survival than distantly related families. The 71 MBP families are the seventh selected MBP generation and are all genetically related through a pedigree [16].

The assumptions for the model were:

$$ \mathbf{y}\sim \mathrm{N}\left(\boldsymbol{\upmu}, {\upsigma}_{\mathrm{a}}^2\mathbf{A}+{\upsigma}_{\mathrm{e}}^2\mathbf{I}\right)\ {\upsigma}_{\mathrm{a}}^2,{\upsigma}_{\mathrm{e}}^2\sim \mathrm{U}\left(0,{\upsigma}_{\mathrm{y}}^2\right) $$

The phenotype **y** was assumed to be distributed as a multivariate normal distribution with the mean **μ** being the mean of **y** and the covariance matrix ${\upsigma}_{\mathrm{a}}^2\mathbf{A}+{\upsigma}_{\mathrm{e}}^2\mathbf{I}$, where **A** was the additive relationship matrix [23], ${\upsigma}_{\mathrm{a}}^2$ was the additive genetic variance, **I** was an identity matrix, and ${\upsigma}_{\mathrm{e}}^2$ was the error variance. The additive relationship matrix was created using the relatedness of the current generation of oysters with the preceding six generations. The variances were assumed to be distributed as uniform distributions bound between zero and the variance of **y**. The posterior distributions of the variances were obtained through Markov chain Monte Carlo (MCMC) sampling using the No-U-Turn sampler implemented in Stan [24]. Four Markov chains were generated with 10,000 iterations per chain, half of which were warmup iterations. Convergence of the Markov chains to the posterior variance distributions was checked by observing if the Gelman-Rubin statistic was close to one [25]. The 20,000 post-warmup samples were used to obtain posterior modes and 95% highest posterior density intervals [22] for the variance and heritability estimates. Heritability (h^2^) was calculated as $\frac{\upsigma_{\mathrm{a}}^2}{\upsigma_{\mathrm{a}}^2+\frac{\upsigma_{\mathrm{e}}^2}{\mathrm{n}}}$ where n was the number of replicates, which was two in the plate assay and four in the field trial. A parameter was considered significant if its 95% highest posterior density interval did not include zero.

To determine if survival values to the French OsHV-1 μvar (C-25) and the Tomales Bay (C-89) OsHV-1 variant were correlated, 95% confidence intervals for Pearson correlation coefficients between breeding values of phenotypes were calculated to determine significant correlations, which were correlations whose confidence intervals did not contain zero, from the Fisher transformation [26]. Breeding values of phenotypes were obtained from the equation $\boldsymbol{\upmu} +{\upsigma}_{\mathrm{a}}^2\mathbf{A}{\left({\upsigma}_{\mathrm{a}}^2\mathbf{A}+{\upsigma}_{\mathrm{e}}^2\mathbf{I}\right)}^{-\mathbf{1}}\left(\mathbf{y}-\boldsymbol{\upmu} \right)$, Because not all 71 MBP families were tested in both experiments, breeding values of families missing in either experiment were predicted by adding their relationships to the non-missing families as rows in the left-hand side **A** of the above equation.

## Results

### Plate assay

**Fig. 1:**
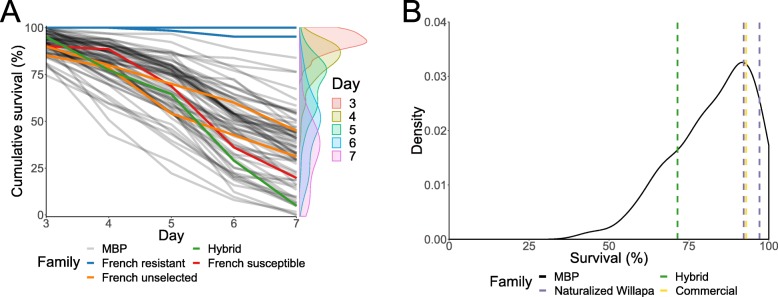
**a** Mean cumulative percent survival of 69 MBP families, one hybrid family, two French selected resistant families, one French selected susceptible family, and two French unselected families from day three post-infection with the French OsHV-1 variant (C-25) to day seven in a plate assay in France with two replications. Distributions of the mean cumulative percent survival are shown on the right-hand side. **b** Distribution of the mean percent survival of 66 MBP families replicated four times after a significant OsHV-1 (C-89) mortality event in Tomales Bay, California with the mean percent survival of one hybrid family, two naturalized Willapa families, and one commercial family shown by dashed vertical lines

**Fig. 2:**
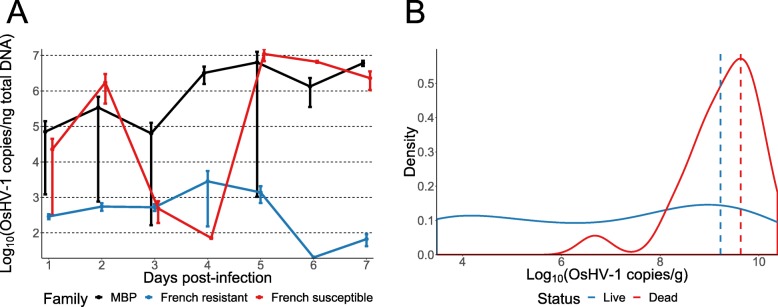
**a** Logarithm-transformed means and standard errors of OsHV-1 variant DNA concentration measured using qPCR over time from pooled spat (*n* = 3 pools, 5 spat per pool) in the plate assay for five random MBP families, two French selected resistant families, and one French selected susceptible family. No viral copies were detected at the start of the experiment (not shown due to log transformation). At day six, the OsHV-1 DNA concentrations (untransformed scale) of the French selected resistant families and the MBP families, which were not significantly different from each other (*p* > 0.05), were significantly less (*p* < 0.05) than the French selected susceptible family. At day seven, the MBP families had a significantly greater OsHV-1 DNA concentration than the French selected resistant families while the French selected susceptible family had an OsHV-1 DNA concentration that was not significantly different than either of the two other groups of families. No other within-day group comparisons were statistically significant in the untransformed scale. **b** Logarithm-transformed OsHV-1 DNA concentration distributions for live (*n* = 26) and dead (*n* = 22) random MBP oysters that were sampled on 1 August 2018 from Tomales Bay and tested positive for OsHV-1 by qPCR. Five live oysters tested negative for OsHV-1 (not shown due to log transformation) and one dead oyster has no amplifiable DNA. Logarithm-transformed means of live (OsHV-1 negative and positive) and dead oysters are shown by dashed vertical lines

Only a single oyster in the control treatment died among all MBP, hybrid, and French families tested. The distribution for the mean cumulative survival in the viral exposure treatment on day three was truncated normal and on days four to seven were approximately normal, with mean cumulative survival decreasing over time (Fig. 1a). By day seven, the two French selected resistant families had a higher cumulative survival (100 and 95%) than the two French unselected families (45 and 30%) and the French selected susceptible family (21%). The hybrid family had a cumulative survival (5%) that was greater than four MBP families out of the 69 MBP families tested. The mean cumulative survival of the MBP families was 34% and varied greatly from 0 to 84%. The mean absolute difference in percent survival among replicates of the MBP families was 6, 10, 14, 18, and 17%, respectively, for days three to seven. No viral DNA was detected in spat from the MBP or French families at the beginning of the plate assay. Viral DNA concentrations increased over the span of the experiment in the five random MBP and French selected susceptible families with the French selected resistant families having significantly lower viral DNA than MBP families by day seven (Fig. 2a). The mean cumulative survival of the five random MBP families was the same as that of all the MBP families (34%). The cumulative survival phenotypes for each time point were significantly heritable, with the highest heritability at five days post-infection (0.69) and the lowest heritability at three days post-infection (0.57) (Table 1). The breeding values between any two time points were significantly positively correlated (0.59–0.95) (Table 2).

**Table 1:** Posterior modes (95% highest posterior density intervals in parentheses) of Bayesian Gaussian process regression model parameters estimated from the MBP families for the plate assay (PA) across days post-infection and the Tomales Bay field trial (TB). The parameters are the genetic (${\upsigma}_{\mathrm{a}}^2$) and error (${\upsigma}_{\mathrm{e}}^2$) variances. Heritability (h^2^) is a statistic calculated from the variances

Phenotype	${\upsigma}_{\mathrm{a}}^2$	${\upsigma}_{\mathrm{e}}^2$	h^2^
PA Day 3	15.76 (3.42–34.56)	33.13 (24.59–45.76)	0.57 (0.22–0.74)
PA Day 4	81.61 (39.99–141.44)	88.12 (65.03–127.46)	0.68 (0.46–0.80)
PA Day 5	160.46 (82.36–266.01)	162.29 (116.53–226.35)	0.69 (0.49–0.81)
PA Day 6	225.05 (97.14–406.81)	292.37 (205.81–409.70)	0.63 (0.40–0.79)
PA Day 7	229.34 (100.40–415.37)	282.49 (205.72–410.45)	0.65 (0.41–0.79)
TB	79.31 (31.41–160.97)	318.01 (272.47–388.68)	0.53 (0.31–0.70)

**Table 2:** Pearson correlation coefficients (95% confidence intervals in parentheses) between breeding values of phenotypes for the plate assay (PA) across days post-infection and the Tomales Bay field trial (TB)

	PA Day 3	PA Day 4	PA Day 5	PA Day 6	PA Day 7	TB
PA Day 3	1	0.74 (0.61–0.83)	0.67 (0.51–0.78)	0.66 (0.51–0.78)	0.59 (0.41–0.72)	0.24 (0.01–0.45)
PA Day 4		1	0.93 (0.88–0.95)	0.86 (0.78–0.91)	0.79 (0.69–0.87)	0.2 (−0.04–0.41)
PA Day 5			1	0.90 (0.85–0.94)	0.84 (0.75–0.90)	0.17 (−0.06–0.39)
PA Day 6				1	0.95 (0.92–0.97)	0.17 (−0.06–0.39)
PA Day 7					1	0.24 (0.01–0.45)
TB						1

### Field trial

**Fig. 3:**
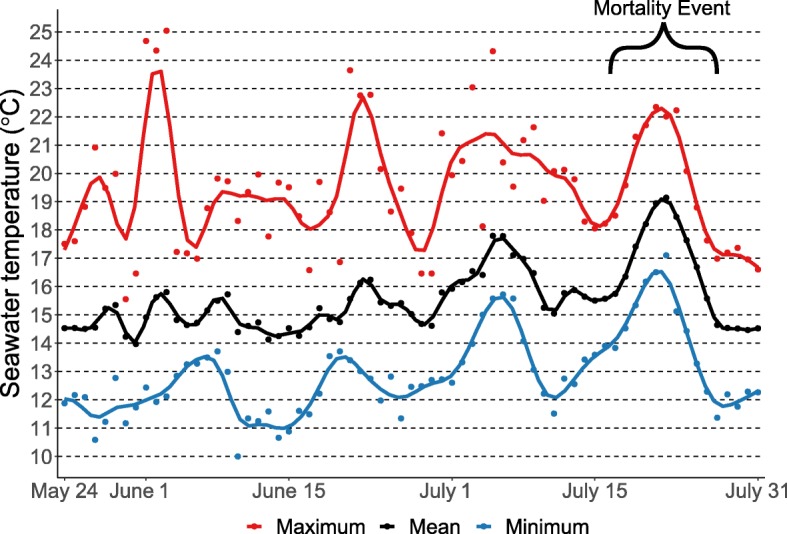
Maximum, mean, and minimum daily seawater temperatures at the Tomales Bay planting site from the day after planting to the day before phenotyping the oysters for survival against the OsHV-1 mortality event plotted as raw values (points) and smoothed lines. Spat were planted on 23 May 2018 and OsHV-1 survival was phenotyped on 1 August 2018

On 1 August 2018, family mortality counts from a significant mortality event coinciding with elevated temperatures (Fig. 3) were recorded and showed a truncated normal distribution (Fig. 1b). The two naturalized Willapa families (97 and 92%) and the commercial family (93%) had higher survival than the hybrid family (71%), which had greater survival than 14 MBP families out of the 66 MBP families tested. The survival phenotype among the MBP families had a mean of 83% and ranged from 45 to 99% and the phenotype was significantly heritable (Table 1). The mean standard deviation among replicates was 18%. All sampled oysters except five live oysters tested positive for OsHV-1 DNA, and all sampled oysters except one dead oyster had amplifiable DNA. Viral DNA concentrations from live oysters sampled were uniformly distributed in the logarithm scale (Fig. 2b) and ranged from zero OsHV-1 copies/g to 2.4 × 10^10^ OsHV-1 copies/g. Viral DNA concentrations from dead oysters sampled were approximately normally distributed in the logarithm scale and ranged from 4.8 × 10^6^ OsHV-1 copies/g to 2.2 × 10^10^ OsHV-1 copies/g. The mean viral DNA concentrations for the live and dead oysters were 1.7 × 10^9^ OsHV-1 copies/g and 4.3 × 10^9^ OsHV-1 copies/g, respectively.

### Correlation between the plate assay and field trial

**Fig. 4:**
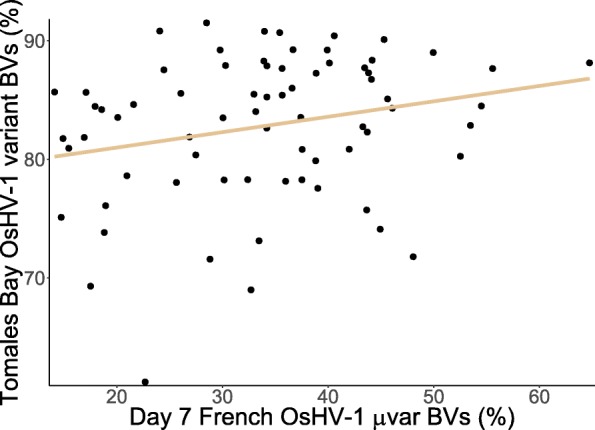
Breeding values (BVs) of MBP families for survival to the French OsHV-1 μvar (C-25) in the plate assay seven days post-infection and to the Tomales Bay OsHV-1 variant (C-89) in the field trial with the line of best fit

A weak but significant correlation of 0.24 was found between the day three plate assay breeding values and the field trial breeding values as well as between the day seven plate assay and the field trial (Table 2; Fig. 4). No other between-experiment correlations were significant.

## Discussion

Significant genetic variation for resistance to two variants of OsHV-1 exists in the MBP breeding population (Table 1), which has not been previously selected for this trait. This finding is similar to the results reported for the French and Australian breeding programs [9, 10], where genetic variation for resistance to OsHV-1 was reported in previously unselected Pacific oysters and used to develop resistant broodstock. Whether the molecular pathways responsible for resistance to OsHV-1 are similar in resistant oysters from these breeding programs remains to be determined.

By using time-series viral DNA quantification in the plate assay and individual oyster OsHV-1 quantification from oysters in Tomales Bay, we confirmed that the mortality observed in both experiments was related to the presence of high loads of OsHV-1 DNA (Fig. 2), although other causes might interact in Tomales Bay. Seawater temperature data suggest that the mass mortality event observed was partly due to mean temperatures reaching 18 °C and above (Fig. 3), which is lower than the constant 20 °C experienced by spat in the laboratory plate assay. Mortality due to OsHV-1 in the outer bay of Tomales Bay, where the oysters were planted in this study, has previously been reported to be correlated to temperatures above 16 °C [1]. The French resistant families managed to limit infection by the OsHV-1 μvar in comparison to the MBP families or the French susceptible family (Fig. 2a), confirming the previously reported resistance in the French-bred resistant families [27–29]. Mean survival among MBP families in the Tomales Bay field trial was high, as was the variance in survival among families (Fig. 1). Consideration of OsHV-1 survival in Tomales Bay during commercial broodstock selection should increase the mean survival of oysters in the resulting families as the survival variance was partly genetic (Table 1). While survival of the MBP families was high in this field trial, it reflects a single mortality event and the single count did not allow for a final mortality count; however, the plate assay results suggest that obtaining multiple mortality counts over time would not greatly alter this estimate of trait heritability. Furthermore, OsHV-1 survival is partly a function of temperature, so a longer elevated temperature event within permissive temperatures would likely cause greater mortality [1, 30, 31]. If strong genetic correlations do not exist between OsHV-1 survival influenced by temperature events of varying severity, field trials would be an ineffective resistance screening method as oyster growers desire OsHV-1 survival to the worst-case scenario. Additionally, other factors possibly influencing OsHV-1 survival, such as *Vibrio* spp. [27], should be considered in future studies to ensure that selected broodstock are resistant to OsHV-1 across a broad range of environments on the West Coast.

Surprisingly, the genetic correlations between resistance to a French OsHV-1 μvar (C-25) and resistance to the Tomales Bay OsHV-1 variant (C-89) were weak or non-significant (Table 2). An explanation for this might be that only some pathways are effective against particular OsHV-1 variants due to evolution by the virus. It has been reported that oysters become more resistant to OsHV-1 as they age [32, 33], so an interaction between genotype and age might account for the low correlation as the spat in the plate assay were three months old when exposed to OsHV-1 while the spat in the field trial were five months old when exposed to OsHV-1 in combination with an elevated temperature event. Testing both variants in the laboratory with spat fertilized on the same date would determine if the cross-variant correlation deviates significantly from one. If the correlation does not deviate significantly from one, it would be advantageous to continue to test families at multiple ages to exploit the genotype-by-age effect such that oyster families that have high OsHV-1 survival at more than one stage of maturity can be selected. This selection would eliminate the need to plant older oysters on the West Coast to avoid OsHV-1 mortality. Despite the low genetic cross-variant correlations, MBP families with high survival breeding values to both variants exist (Fig. 4) and can be used as parents in future generations.

## Conclusions

This study is the first large-scale screening of Pacific oyster families from the West Coast of the United States for resistance to two variants of OsHV-1. We found that the MBP families have a significant amount of genetic variation in resistance to both the Tomales Bay OsHV-1 variant and the French OsHV-1 μvar. This variation can be exploited through family-based selection to develop resistant broodstock; however, selection for survival to one variant may not greatly increase survival to the other variant due to a low observed correlation between the survival breeding values in the MBP population.

## Data Availability

The data and code used to run the analyses are available at https://github.com/kdivilov/BMC_Genetics_2019.

## Abbreviations

MBP: Molluscan Broodstock Program
MCMC: Markov chain Monte Carlo
OsHV-1: *Ostreid herpesvirus 1*
PA: Plate assay; TB: Tomales Bay
μvar: Microvariant
